# Investigation of Platelet Apoptosis in Patients after Surgical Myocardial Revascularization

**DOI:** 10.3390/biomedicines11020251

**Published:** 2023-01-18

**Authors:** Alisa A. Sokolovskaya, Mikhail A. Popov, Ekaterina A. Sergeeva, Arkadiy A. Metelkin, Dmitry I. Zybin, Dmitry V. Shumakov, Aslan A. Kubatiev

**Affiliations:** 1 Department of Molecular and Cellular Pathophysiology, Research Institute of General Pathology and Pathophysiology, Baltiyskaya 8, 125315 Moscow, Russia; 2 Department of Cardiosurgery, Vladimirsky Moscow Regional Research Clinical Institute, Shepkina 61/2, 129110 Moscow, Russia

**Keywords:** apoptosis, platelets, cardiovascular disease, mitochondria, coronary heart disease

## Abstract

Platelets are one of the main participants in vascular accidents in cases of coronary heart disease (CHD). In this study, we sought to detect platelet apoptosis in patients with coronary artery disease who underwent scheduled myocardial revascularization surgery. To identify apoptotic events, we analyzed phosphatidylserine (PS) expression on the surface of platelets and mitochondrial membrane potential (ΔΨm) by flow cytometry in two groups of 30 patients aged 45–60 years: Group 1—patients before myocardial revascularization surgery and group 2—patients after myocardial revascularization surgery. The control group consisted of 10 healthy volunteers aged 45–60 years. According to our data, the percentage levels of PS expression in patients greatly decreased after surgery. We confirmed platelet apoptosis by recording depolarization of ΔΨm in pre- and postoperative patients. ΔΨm readings were considerably improved after surgery. Our data indicated that the functional parameters of platelets in patients with coronary heart disease differed from the characteristics of platelets in patients who underwent myocardial revascularization, and from those of patients in a control group. Future studies of platelet phenotypic characteristics and platelet apoptosis biomarkers should greatly advance our understanding of the pathophysiology of coronary heart disease, and further promote the development of methods for predicting adverse outcomes after surgery.

## 1. Introduction

Platelets are one of the main participants in vascular accidents in cases of coronary heart disease (CHD).

Platelets play a critical role in thrombotic vascular occlusion at the site of coronary atherosclerotic plaque rupture, leading to acute coronary syndromes (ACSs). Both embolization of platelet aggregates and direct receptor-mediated adhesion of platelets to the surface of postischemic microvessels leads to obstruction and disruption of coronary microcirculation. Such disturbances result in additional tissue damage and exacerbate myocardial contractile dysfunction. In addition, platelets not only contribute to acute thrombotic vascular occlusion, but also participate in the inflammatory and matrix-degrading processes of coronary atherosclerosis itself [1].

Platelets activation leads not only to the formation of a thrombus—a morphological substrate of atherothrombosis—but also to the formation of a large number of biologically active substances. These include growth-promoting substances such as platelet-derived epidermal growth factor (PD-EGF), platelet growth factor (PDGF A + B), transforming growth factor (TGF-β), insulin-like growth factors (IGF-I, II), vascular endothelial growth factor (VEGF), endothelial cell growth factor (ECGF), basic fibroblast growth factor (bFGF), and epidermal growth factor (EGF) [2,3,4].

Other active substances formed include adhesive proteins (fibrinogen, fibronectin, vitronectin, thrombospondin-1), coagulation factors (factor V, factor XI, protein S, antithrombin), fibrinolytic factors (plasminogen, urokinase inhibitor, α2-antiplasmin), proteases (tissue inhibitor of matrix metalloprotease-4), antiproteases (metalloprotease-4, α2-antitrypsin), basic proteins (platelet factor-4, β-thromboglobulin, endostatins), and membrane glycoproteins (CD-40, P-selectin), as well as biologically active molecules contained in dense granules of platelets (serotonin, histamine, dopamine, ADP, ATP, Ca^2+^, catecholamines) [5,6,7,8,9].

Due to such multifunctionality, these cells play key roles both in the hemostasis system and in the regulation of reparative processes. In addition, ischemia/reperfusion-induced alterations in the regulation of cytosolic osmolality and cell volume cause cellular and interstitial edema that are associated with microvascular obstruction, cell dysfunction and death [10].

Apoptosis, or programmed cell death, is a well-studied phenomenon affecting nucleus-containing cells. In the last two decades, researchers have discovered that cytoplasts—non-nucleated cells that are, primarily, erythrocytes [11]—and platelets [12,13] also experience apoptosis.

A decisive role in platelet apoptosis control is played by intracellular events. These include depolarization of mitochondrial membrane potential (ΔΨm); the externalization of phosphatidylserine (PS) on the outer surface of platelets; the activation of caspases 3, 9, and 8; and the activation and translocation of proapoptotic proteins Bax and Bak into mitochondria [14,15].

PS externalization on the outer side of the plasmolemma can be detected using the anticoagulant protein annexin V. This protein is used for the detection of apoptotic cells by means of fluorescence microscopy or flow cytometry [16,17].

The presence of PS on the membranes of activated platelets accelerates the most important coagulation reactions by several orders of magnitude; consequently, it is essential for hemostasis [18].

Platelet apoptosis in various diseases is now a matter of special interest to researchers [19,20,21]. The authors of [10] found that microembolization and platelet accumulation within the affected microcirculation of the myocardium during late ischemia and reperfusion (IR) leads to secondary tissue damage [10].

Platelets secrete chemokines, which play a role in inflammation and hemostasis. These processes are induced by chemokines that are secreted from activated platelets and stored in their granules. Some small molecules that are activated by platelets can be used as markers to predict the clinical outcome of cardiovascular disease patients [11].

Our understanding of platelets is still far from complete. However, further studies of specific platelet characteristics may lead to improved prognoses and better treatment strategies for patients with coronary heart disease.

One potentially rewarding study area concerns platelet apoptosis. The pathophysiology of coronary artery disease and the role of platelet apoptosis in its research. In this study, we focused on platelet apoptosis in patients with chronic coronary heart disease.

## 2. Materials and Methods

### 2.1. Patient Characteristics

We conducted this study in accordance with the ethical principles of the Declaration of Helsinki of the World Medical Association (1964, 2004). We obtained voluntary informed consent in writing from all patients. The study was approved by the ethics committee of the Moscow Regional Research Clinical Institute (No. 13427-c).

The study involved 30 patients suffering from coronary artery disease. The patients were divided into two groups: Group 1—patients before myocardial revascularization surgery and group 2—patients after myocardial revascularization surgery.

The inclusion criteria were as follows according to the ESC/EACTS Guidelines on Myocardial Revascularization 2018: The presence of hemodynamically significant stenosis of the main coronary arteries; a scheduled operation for surgical myocardial revascularization (CABG); a patient age of 45–65 years; and the obtaining of informed consent. Exclusion criteria covered the presence of any of the following: Acute myocardial infarction; chronic heart failure of functional class III–IV; cancer; and blood diseases. The majority of patients exhibited generalized atherosclerotic disease of the coronary arteries. All patients underwent an examination, which included an assessment of physical status, clinical and biochemical tests, ECG, and echocardiography. We confirmed all diagnoses of coronary heart disease by means of coronary angiography.

The control group consisted of 10 healthy volunteers aged 45–60 years. The criteria for inclusion in this cohort were the absence of cardiovascular pathology, blood diseases, cancer, and taking narcotic drugs or drugs that affect the functional state of platelets.

To exclude the presence of coronary heart disease in the control group, all volunteers were assessed for the pre-test probability of coronary heart disease, adopted in our country as the main screening method for conducting an in-depth examination, which would include invasive methods, including CAG, which corresponds to the current clinical recommendations of the Russian Society of Cardiology, while all volunteers with a risk of coronary heart disease less than 5% was determined (extremely low risk).

The absence of modified and unmodified risk factors for the development of coronary heart disease, such as smoking, obesity, diabetes mellitus, and a burdened hereditary history were also strong arguments in favor of the absence of coronary heart disease. In addition, according to the ECG data, there were no signs of ischemia or metabolic changes in the myocardium. According to echocardiography, the volunteers had no areas of contractility disorder or signs of atherosclerosis of the aorta and coronary arteries.

Blood samples were taken from the cubital veins of patients (~5 mL) using a 5 mL disposable syringe containing sodium citrate (2.5%). Samples were processed no later than 1.5 h after venipuncture.

Blood drawn from patients was carried out on the day before the operation, and on the third day after surgery. Volunteers were sampled once, on the day of their examination.

To begin our study, we measured base parameters of intravascular platelet activation, as well as quantitative and qualitative parameters of platelets, in patients with coronary artery disease who were admitted to the cardiac surgery department for coronary artery bypass grafting. We compared these measurements with data from a group of healthy individuals.

### 2.2. Washed Platelet Preparation

Platelet-rich plasma (PRP) was separated by centrifugation (200× *g*, 10 min). Platelets were pelleted from PRP by centrifugation (800× *g*, 20 min) and re-suspended in HEPES-buffered saline (10 mM HEPES, 135 mM NaCl, 3 mM KCl, 0.34 mM NaH_2_PO4, 1 mM MgCl, 2.6 mM H_2_O, (pH 7.4), supplemented with 0.9 mg/mL D-glucose) at 2 × 10^9^ platelets/mL.

### 2.3. Treatment of Platelets with A23187

A23187 or calcium ionophore, a non-physiological platelet agonist, causes platelet apoptosis events, including PS exposure and ΔΨM depolarization [17].

The obtained platelet suspensions were resuspended in platelet buffer and diluted at 1 × 10^8^ platelets/mL. Platelets in a measured amount of 1 × 10^7^ in 100 µL were incubated with A23187 10 μmol/L (Sigma, St. Louis, MO, USA) at room temperature for 15 min.

### 2.4. Analysis Expression of Platelet Surface Receptor of CD61 by Flow Cytometry

**Figure 1:**
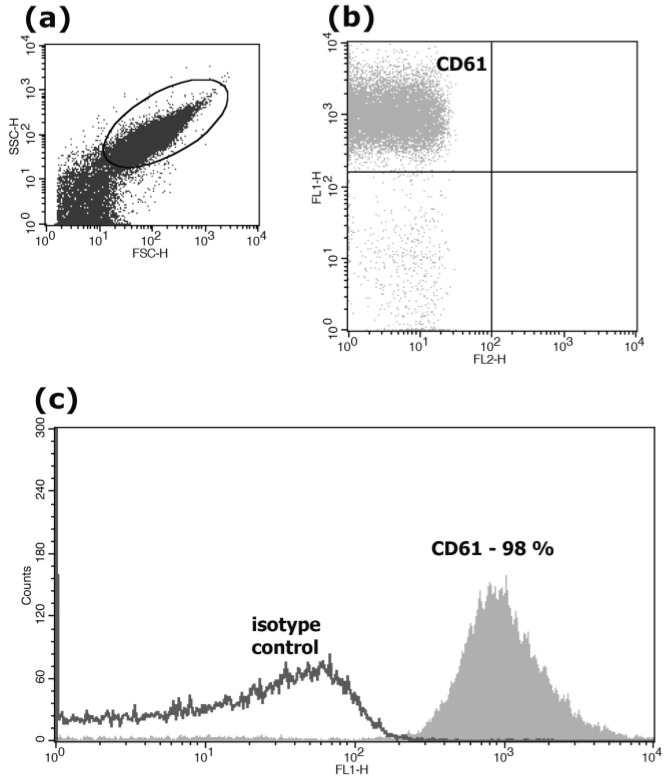
Expression of CD61 on the surface of the patients’ platelets. Sub-figure (**a**) shows cytometric analysis of platelet size distribution (FSC) and granularity (SSC); (**b**) shows staining of platelets with CD61/FITC antibodies; and (**c**) presents a histogram of this experiment, where the *X*-axis represents fluorescence intensity, and the *Y*-axis represents the number of events. We carried out our analysis using a FACSCalibur flow cytometer.

The CD61 antigen (beta III integrin) is present in all normal, resting and activated platelets. The population of platelets was identified on the basis of particle size (forward and 90° side scatter) and the association with CD61. The obtained gated platelets expressed CD61 on the surface in almost 100% of cases (Figure 1).

Platelets in a measured amount of 2 × 10^6^ cells per 100 µL were incubated with monoclonal antibodies for 30 min at 4 °C. Monoclonal antibodies CD61/FITC (BD, Biosciences, USA), were used for characterization. After incubation, 400 µL of citrate buffer was added to the cells and a FACSCalibur flow cytometer (Becton Dickinson, Franklin Lakes, NJ, USA) was used for analysis. Thirty thousand events were accumulated for each sample. Data were collected using the CELLQuest program (Becton Dickinson, Franklin Lakes, NJ, USA).

### 2.5. Phosphatidylserine (PS) Externalization Analysis

After incubation with A23187, platelets in a measured amount of 100 µL (1 × 10^7^) were transferred from the positive control to a tube containing 10 µL of annexin V-FITC in binding buffer (BD Biosciences, Franklin Lakes, NJ, USA). The same procedure was carried out for non-activated platelets. All samples were incubated at room temperature in the dark for 15 min. After that, 400 µL of binding buffer was added, and the sample was immediately analyzed using a FACSCalibur flow cytometer (Becton Dickinson, Franklin Lakes, NJ, USA) equipped with an air-cooled argon laser (wavelength 488 nm). Thirty thousand events were accumulated for each sample. Data were collected using the CELLQuest program (Becton Dickinson, Franklin Lakes, NJ, USA).

### 2.6. Analysis of Mitochondrial Membranes Potential (MMP)

MMP was determined using JC-1 equipment (BD Biosciences, Franklin Lakes, NJ, USA) according to the manufacturer’s instructions. Briefly, JC-1 was dissolved in 500 μL DMSO to obtain stock solution and stored at −20 °C in separated tubes. The stock solution was further diluted in platelet buffer on the day of staining.

After incubation with A23187, platelets (1 × 10^7^) were diluted in 500 μL buffer solution and incubated with 5 μL JC-1 for 15 min in the dark at 37 °C. Platelets were then washed twice with buffer solution. The supernatant was discharged, and 500 μL buffer solution was added. The samples were immediately analyzed using a flow cytometer FACSCalibur (Becton Dickinson, Franklin Lakes, NJ, USA). Fifty thousand events were accumulated for each sample. Data were collected using the CELLQuest program (Becton Dickinson, Franklin Lakes, NJ, USA).

### 2.7. Statistical Processing of Results

Flow cytometry results are presented as arithmetic mean and standard deviation. As all study variables passed the Shapiro–Wilk normality test, we analyzed differences between groups using one-way ANOVA. Repeated measure ANOVA was applied quantitatively for parameters of platelets in pre- and postoperative patients (*p* > 0.05). All statistical analyses were carried out using Statistica for Windows.

## 3. Results

### Subsection

We analyzed changes in the parameters of intravascular platelet activation on the third day after surgery. When we compared the quantitative indicators of platelets in pre- and postoperative patients, we found no significant decreases in platelet counts after surgery.

To detect apoptotic cells, we used annexin V and flow cytometry.

**Figure 2:**
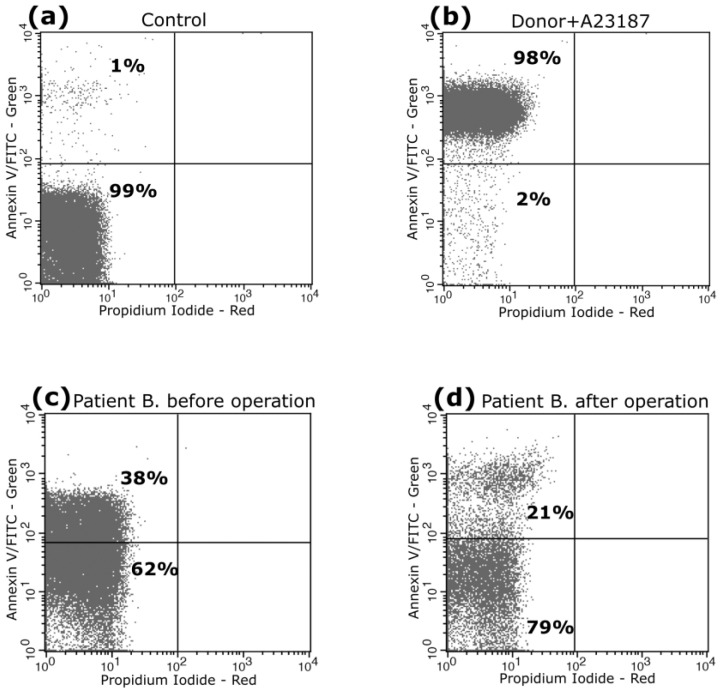
Exposure of phosphatidylserine on the surface of A23187-treated platelets. Representative dot plots are shown for platelets from a healthy donor treated with control diluent buffer (**a**) and A23187 (**b**). The numbers in the upper-left quadrant show the average percentage of annexin V-positive cells with green (FL1) fluorescence in the group of buffer-treated platelets. Externalization of PS on platelets of patient B before surgery are shown in (**c**) and platelets of the same patient after surgery are shown in (**d**). Red (FL2) fluorescence (propidium iodide) revealed no cells in late-stage apoptosis or necrosis, as illustrated by the empty lower-right quadrants of the figure. We carried out our analysis using a FACSCalibur flow cytometer.

**Figure 3:**
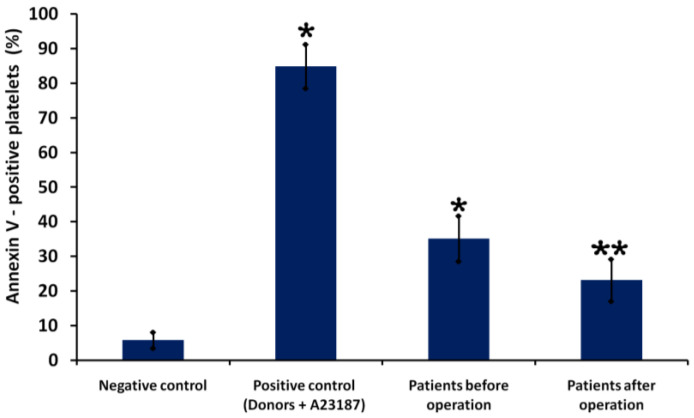
Quantitative parameters of platelet PS externalization in patients before and after surgery. Platelets from a healthy donor treated with control diluent buffer (negative control) and A23187 (positive control). Results are representative from 30 patients and 10 healthy donors. * indicates a significant difference vs. control (healthy donors) (*p* < 0.01); **—indicates a significant difference before and after surgery (*p* < 0.05, *n* = 30).

Figure 2 shows fluorometric parameters of PS externalization in platelets of Patient B before (38%) and after surgery (21%). We found that levels of PS expression on the platelets of patients overall were significantly higher before surgery (35 ± 6%) than after surgery (23 ± 5%) (*p* < 0.05, *n* = 30). Figure 3 illustrates quantitative parameters of PS externalization in patients’ platelets before and after surgery.

We further determined the loss of ΔΨm as an indicator of apoptosis. ΔΨm is another important factor in platelet apoptosis events. According to some reports, the formation of JC-1 aggregates is very sensitive to temperature changes, so we used a method in which platelets were incubated at a temperature of 37 °C for 30 min [22]. The principle of the method can be stated: In the presence of an increased membrane potential, the JC-1 dye accumulates in the form of J-aggregates in the matrix of polarized mitochondria that are recorded by the red fluorescence channel (FL2). During mitochondrial depolarization, JC-1 does not accumulate inside the membrane. It forms monomeric forms, which are registered by the green fluorescence channel (FL1). There is, therefore, a change in fluorescence from red (FL2) to green (FL1), which can be confirmed by flow cytometry.

**Figure 4:**
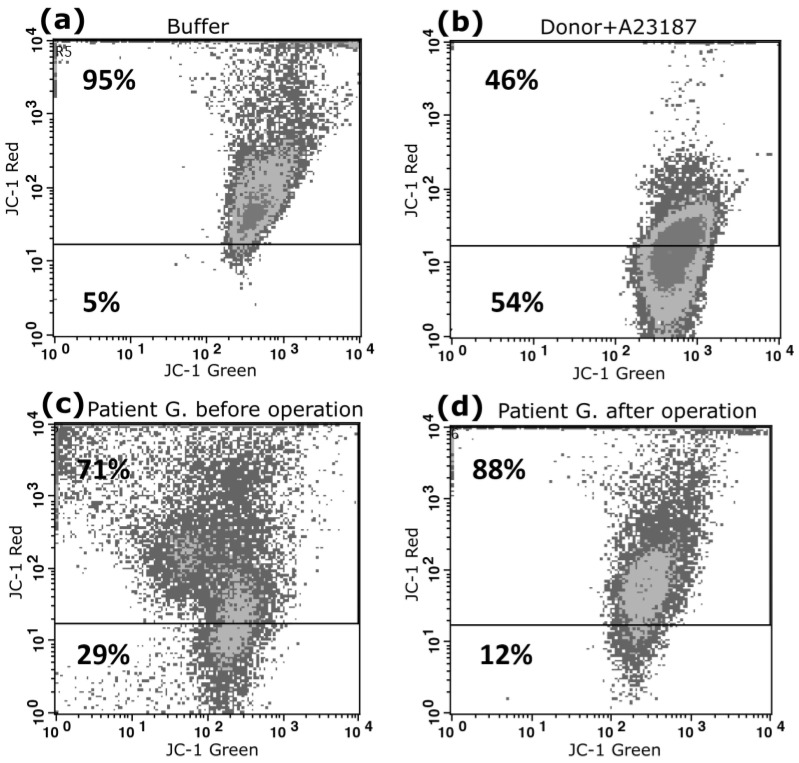
Cytofluorometric analysis of ΔΨm of platelets stained by JC-1. Representative dot plots are shown for the following: platelets from a healthy donor treated with control diluent buffer (**a**) and A23187 (**b**); ΔΨm platelets of patient B before surgery (**c**) and after surgery (**d**). Depolarization is characterized as the decrease in the content of JC-1 aggregates, as reflected in the decrease in red (FL2) fluorescence. Horizontal lines indicate the mean percentage of cells undergoing ΔΨm depolarization in the buffer-treated platelet group. Numbers in the lower parts of dot plots represent percentages of ΔΨm-depolarized cells. We carried out our analysis using a FACSCalibur flow cytometer.

Figure 4 presents cytometric dot plots of platelets showing JC-1 staining. In patient G, before surgery, we recorded membrane depolarization in 29% of platelets (Figure 4c); after the operation, the corresponding figure was 12% (Figure 4d). As a positive control, we recorded membrane depolarization changes in the platelets of a healthy donor, which were activated with a calcium ionophore (Figure 4b). We found that almost 54% of platelets were depolarized under the influence of calcium ionophore.

**Figure 5:**
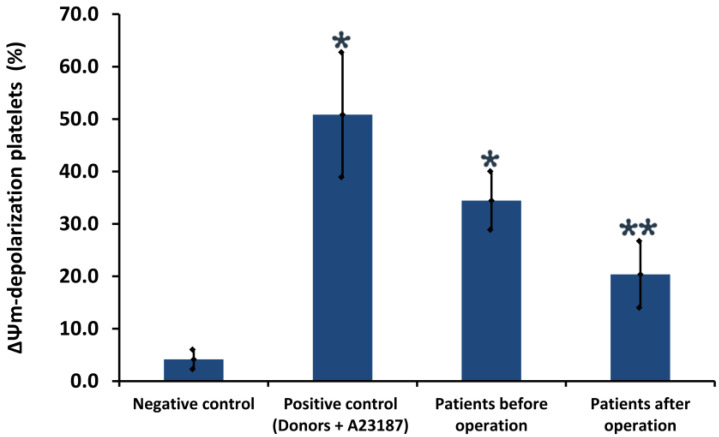
Quantitative parameters of mitochondrial membrane potential (ΔΨm) in platelets. Platelets from a healthy donor treated with control diluent buffer (negative control) and A23187 (positive control). Results are representatives from 10 healthy donors and 30 patients. * indicates a significant difference vs. healthy donors (*p* < 0.05); ** indicates a significant difference before and after surgery (*p* < 0.05, *n* = 30).

Figure 5 illustrates quantitative parameters of ΔΨm in patients’ platelets before and after surgery, of 33.5 ± 5.6% and 20.3 ± 5.3%, respectively (*p* < 0.05, *n* = 30).

Thus, platelet depolarization ΔΨm may reflect the severity of the disease in preoperative patients; however, after coronary artery bypass grafting, ΔΨm indicators change for the better with statistically significant differences between pre- and post-operative groups.

In this study, we demonstrated that reduced platelet PS and depolarization of the mitochondrial membrane are closely associated with myocardial revascularization.

## 4. Discussion

According to the literature, many studies demonstrate that inflammatory mechanisms play a key role in the pathophysiology of reperfusion. Currently, thrombotic occlusion can be eliminated through the timely use of fibrinolytic drugs or myocardial revascularization. Revascularization of the coronary blood flow is a necessary condition for the restoration of adequate metabolism in the ischemic area of the myocardium.

However, after successful revascularization, the restoration of the function of the ischemic area of the myocardium depends on its safety and the development of microcirculation in this area. Restoration of blood flow in the ischemic myocardium manifests itself as an acute inflammatory response, including the secretion of cytokines, the expression of cell adhesion molecules, neutrophilic infiltration, and increased microvascular permeability [23].

The pathological role of platelets in the inflammatory response has been studied. Activated platelets attach to the endothelium and leukocytes, causing an inflammatory response.

Platelets protect the myocardium from ischemia/reperfusion-induced injury, and these protective effects of platelets are evident regardless of the duration of ischemia/reperfusion [24].

The hemostatic and prothrombotic functions of platelets are critically dependent on two key properties: (1) Their ability to attach and aggregate at the sites of vascular injury; and (2) their ability to maintain the blood clotting required for thrombin and fibrin (platelet procoagulant) formation. The transformation of activated platelets into a procoagulant state is associated with specific biochemical and morphological changes, some of which are similar to those occurring in apoptotic cells, including caspase activation, proteolytic processing of cytoskeletal elements, and surface exposure to PS, as well as membrane contraction, blebbing, and vesicle formation [25].

The expression of the activation markers CD62P and CD63 increases considerably during activation but not during apoptosis. In contrast, the correlation between annexin V and CD62P expression during apoptosis (or late activation) suggests a link between platelet activation and apoptosis. Some researchers have suggested that externalization of PS may exhibit platelet apoptosis earlier than platelet activation [26].

In this study, we used CD62P to determine platelet activation. According to our preliminary data, the percentage of platelets expressing CD62P was higher in preoperative patients. We found no significant correlation between platelet activation and other parameters in patients before and after coronary artery bypass grafting.

While platelets do not possess a nucleus, they do possess mitochondria, and these play an important role in platelet activation by providing ATP energy [27,28]. Previous studies have reported mitochondrion-mediated platelet apoptosis [28,29].

Various agonists cause apoptotic events in platelets. These include membrane depolarization and activation of caspases, especially caspase 3. However, PS expression only occurs during platelet apoptosis under strong agonist conditions [28,29].

While agonist-induced procoagulant PS platelets exhibit the characteristics of apoptotic cells, the processes which regulate their formation are distinct from those regulating platelet apoptosis [30].

The use of flow cytometry has become widespread in the fields of hemostasis and thrombosis because of its practical advantages over time-consuming and expensive conventional platelet assays such as enzyme-linked immunosorbent assay (ELISA), transmission electron microscopy (TEM), high-performance liquid chromatography (HPLC), aggregometry, and radioimmunoassay [31]. In flow cytometry, individual cells are passed through a laser beam so that light scatter (indicating cell size and granularity) and signal intensity from various fluorescently labeled cell-associated markers can be measured. This enables the study of different aspects of platelet function in response to various platelet agonists [32].

Platelet activation, as identified by increased expression of PS and P-selectin, has been observed in heparin-induced thrombocytopenia (HIT). This immune activation may be accompanied by biochemical signs of apoptosis such as mitochondrial membrane depolarization, Bax activation, Bcl-XL negative regulation, procaspase 3 activation, and increased calpain activity [31,32,33,34].

Some recent studies have suggested that mitochondrial platelet metabolism is involved in the mechanism of thrombocytopenia in preeclampsia. Platelet ΔΨm can serve as a prognostic marker in stratifying clinical risk and also as a gauge of disease activity which may prove valuable in future studies [35].

Other studies have highlighted a potential prognostic role for flow-cytometric assessment of ΔΨm in measuring apoptosis of circulating monocyte–platelet aggregates in patients with symptomatic ischemic heart disease [36].

In this study, to identify apoptotic events, we analyzed PS expression on the surfaces of patient platelets using the flow cytometry method. According to our data, the percentage of PS expression decreased after surgery. We also confirmed platelet apoptosis by recording depolarization of ΔΨm in patients before and after surgery. In postoperative patients, ΔΨm values returned to normal values.

These changes in the ΔΨm were associated with an improvement in myocardial contractility according to instrumental data, as well as the clinical condition of patients with positive dynamics after surgery. All patients had multivessel coronary artery disease. The severity of coronary stenosis was more than 75% of the main arteries.

In our opinion, after myocardial revascularization in patients with coronary heart disease, there is an improvement in the contractile function of the myocardium, and, consequently, a decrease in global ischemia. There is an improvement in both coronary and regional blood flow, and in particular, at the level of blood cells—platelets.

At the same time, after revascularization, there is an increase in the inflammatory response, in which the role of platelets has a separate place, however, against the background of taking antiplatelet agents, a partial deactivation of inflammatory cytokines occurs, and therefore the risk of repeated thrombosis decreases.

Based on the data presented here, we can assume that the degree of influence of myocardial revascularization factors brings about a corresponding improvement in cardiac output. The time factor—the three-day period during which there is a significant decrease in apoptosis—deserves special attention in future research.

In summary, despite the limited number of patients, our data indicate that the functional parameters of platelets in patients with coronary heart disease differ from those in patients after myocardial revascularization, as well as those in the control group. Larger-scale studies are needed to determine the potential prognostic role of flow-cytometric evaluation of ΔΨm as well as other markers of apoptosis in circulating aggregates in patients with coronary heart disease.

## 5. Conclusions

Future studies of platelet phenotypic characteristics and platelet apoptosis biomarkers should greatly advance our understanding of the pathophysiology of coronary heart disease, and further promote the development of methods for predicting adverse outcomes after surgery.

## Data Availability

The raw data used in this study are available upon request from the corresponding author.

## Abbreviations

Abs	Antibodies
ACD	Acid-citratedextrose
ACS	Acute coronary syndrome
ATP	Adenosine triphosphate
bFGF	basic fibroblast growth factor
CD61	Cluster of Differentiation 61
CHD	coronary heart disease
DTT	Dithiothreitol
ECGF	endothelial cell growth factor
EGF	epidermal growth factor
FITC	Fluorescein isothiocyanate
HIT	heparin-induced thrombocytopenia
IGF-I, II	insulin-like growth factors
JC-1	(5,5′,6,6′-Tetrachloro-1,1′,3,3′-tetraethylbenzimidazolylcarbocyanine, iodide)
PD-EGF	Platelet-derived epidermal growth factor
PDGF A + B	platelet growth factor
PI	Propidium-iodide
PRP	Platelet-rich plasma
PS	Phosphatidylserine
RT	Room temperature
TGF-β	transforming growth factor
ΔΨm	mitochondrial membranes potential

## References

[1] Massberg S., Schulz C., Gawaz M. (2003). Role of Platelets in the Pathophysiology of Acute Coronary Syndrome. Semin. Vasc. Med..

[2] Bao J., Lin L. (2014). Platelet apoptosis in patients with acute coronary syndromes. J. Thromb. Thrombolysis.

[3] Andrae J., Gallini R., Betsholtz C. (2008). Role of platelet-derived growth factors in physiology and medicine. Genes Dev..

[4] Gabbasov Z.A., Ryzhkova E.V. (2014). Platelet phenotype in myocardial infarction. Kreat. Kardiol..

[5] Järemo P., Eriksson M., Lindahl T.L., Nilsson S., Milovanovic M. (2012). Platelets and acute cerebral infarction. Platelets.

[6] Clemetson K.J., Clemetson J.M. (2013). Platelet Receptors. Platelets.

[7] Blann A.D., Nadar S.K., Lip G.Y. (2003). The adhesion molecule P-selectin and cardiovascular disease. Eur. Heart J..

[8] Gavaz M. (2004). Role of platelets in coronary thrombosis and reperfusion of ischemic myocardium. Cardiovasc. Res..

[9] Khodadi E. (2020). Platelet Function in Cardiovascular Disease: Activation of Molecules and Activation by Molecules. Cardiovasc. Toxicol..

[10] Schanze N., Bode C., Duerschmied D. (2019). Platelet Contributions to Myocardial Ischemia/Reperfusion Injury. Front. Immunol..

[11] Bratosin D., Estaquier J., Ameisen J.C., Montreuil J. (2002). Molecular and cellular mechanisms of erythrocyte programmed cell deach: Impact on blood transfusion. Vox Sang..

[12] Mindukshev I.V., Rukoyatkina N.I., Dobrylko I.A., Skvertchinskaya E.A., Nikitina E.R., Krivoshlyk V.V., Gambaryan S.P., Krivchenko A.I. (2013). Characterisation of enucleated cells apoptosis: Human platelets and erythrocytes. Ossiiskii Fiziol. Zhurnal Im. IM Sechenova.

[13] Brown S.B., Clarke M., Magowan L., Sanderson H., Savill J. (2000). Constitutive Death of Platelets Leading to Scavenger Receptor-mediated Phagocytosis. A caspase-independent cell clearance program. J. Biol. Chem..

[14] Mason K.D., Carpinelli M.R., Fletcher J.I., Collinge J.E., Hilton A.A., Ellis S., Kelly P.N., Ekert P.G., Metcalf D., Roberts A.W. (2007). Programmed Anuclear Cell Death Delimits Platelet Life Span. Cell.

[15] Leytin V. (2012). Apoptosis in the anucleate platelet. Blood Rev..

[16] Josefsson E., White M., Dowling M., Kile B.T. (2012). Platelet Life Span and Apoptosis. Methods Mol. Biol..

[17] Båla N., Debreceni I.B., Kappelmayer J. (2013). Flow Cytometric Investigation of Classical and Alternative Platelet Ac-tivation Markers. EJIFCC.

[18] Ozge C. (2013). The apoptotic actions of platelets in acute ischemic stroke. Mol. Biol. Rep..

[19] Thushara R.M., Hemshekhar M., Basappa, Kemparaju K., Rangappa K.S. (2015). Platelet apoptosis and human diseases: An outlook. Crit. Rev. Oncol..

[20] Stein T., Hinselmann R., Gowin C., Greber D., Shllaku S., Gölz N., Schmugge M., Franzoso F.D. (2022). Activation of Platelet Apoptosis and Autophagy in Immune Thrombocytopenia: New Mechanistic Insights. J. Comp. Pathol..

[21] Wu F., Liu Y., Luo L., Lu Y., Yew D.T., Xu J. (2015). Platelet mitochondrial dysfunction of DM rats and DM patients. Int. J. Clin. Exp. Med..

[22] Verhoeven A.J., Robin V., Eric G.W., Gouwerok and Dirk de Korte (2005). The mitochondrial membrane potential in human platelets: A sensitive parameter for platelet quality. Transfusion.

[23] Hansen P.R. (1998). Inflammatory Alterations in the Myocardial Microcirculation. J. Mol. Cell. Cardiol..

[24] Yang B.C., Virmani R., Nichols W.W., Mehta J.L. (1993). Platelets protect against myocardial dysfunction and injury induced by ischemia and reperfusion in isolated rat hearts. Circ. Res..

[25] Dale G.L. (2005). Coated-platelets: An emerging component of the procoagulant response. Thromb. Haemost..

[26] Unsal O., Ozerol E., Mehmet A. (2007). Relationship between activation and apoptosis in platelets. Turk. J. Hematol..

[27] Zhang W., Liu J., Sun R., Zhao L., Du J., Ruan C. (2011). Calpain Activator Dibucaine Induces Platelet Apoptosis. Int. J. Mol. Sci..

[28] Zhang W., Zhao L., Liu J., Du J., Wang Z., Ruan C., Dai K. (2012). Cisplatin induces platelet apoptosis through the ERK signaling pathway. Thromb. Res..

[29] Leytin V., Allen D.J., Mykhaylov S., Mis L., Lyubimov E.V., Garvey B. (2004). Pathologic high shear stress induces apoptosis events in human platelets. Biochem. Biophys. Res. Commun..

[30] Schoenwaelder S.M., Yuan Y., Josefsson E.C., White M.J., Yao Y., Mason K.D., O’Reilly L.A., Henley K.J., Ono A., Hsiao S. (2009). Two distinct pathways regulate platelet phosphatidylserine exposure and procoagulant function. Blood.

[31] Carubbi C., Masselli E., Vitale M., Gresele P., Kleiman N., Lopez J., Page C. (2017). Flow Cytometry. Platelets in Thrombotic and Non-Thrombotic Disorders.

[32] Ramström S., Södergren A., Tynngård N., Lindahl T. (2016). Platelet Function Determined by Flow Cytometry: New Perspectives?. Semin. Thromb. Hemost..

[33] Ferroni P., Pulcinelli F.M., Lenti L., Gazzaniga P.P. (1999). Is soluble P selectin determination a more reliable marker of in vivo platelet activation than CD62P flow cytometric analysis?. Thromb Haemost..

[34] Mordakhanova E.R., Nevzorova T.A., Synbulatova G.E., Rauova L., Weisel J.W., Litvinov R.I. (2020). Platelet Activation in Heparin-Induced Thrombocytopenia is Followed by Platelet Death via Complex Apoptotic and Non-Apoptotic Pathways. Int. J. Mol. Sci..

[35] Kraemer B.F., Hennis I., Karge A. (2022). Platelet mitochondrial membrane depolarization reflects disease severity in patients with preeclampsia. Mol. Med..

[36] Vogel S., Rath D., Lu J., Chatterjee M., Geisler T., Gawaz M. (2015). Elevated mitochondrial membrane potential of circulating monocyte–platelet aggregates in patients with coronary heart disease. Int. J. Cardiol..

